# From stress signals to fertility challenges: the role of damage-associated molecular patterns in male reproduction

**DOI:** 10.3389/fimmu.2025.1598451

**Published:** 2025-10-01

**Authors:** Heran Cao, Shujuan Liu, Shichao Cui, Hua Nie, Xiaohua Liu, Weibing Qin

**Affiliations:** ^1^ The NHC Key Laboratory of Male Reproduction and Genetics, Family Planning Research Institute of Guangdong Province, Guangzhou, China; ^2^ Department of Central Laboratory, Guangdong Provincial Reproductive Science Institute (Guangdong Provincial Fertility Hospital), Guangzhou, China; ^3^ College of Animal Science and Technology, Northwest A&F University, Xianyang, Shaanxi, China

**Keywords:** male infertility, DAMPs, spermatogenesis, sperm maturation, fertilization, inflammatory response

## Abstract

Male infertility is influenced by genetic abnormalities, hormonal imbalances, lifestyle factors, and environmental exposures. Recently, Damage-Associated Molecular Patterns (DAMPs) have emerged as key players in male reproductive health, particularly in regulating inflammatory responses and tissue damage. This review highlights the role of critical DAMPs, such as HMGB1, HSPs, ATP, eCIRP, histones, and cfDNA, in processes like spermatogenesis, sperm maturation, and fertilization. Released through mechanisms like necrosis, apoptosis, pyroptosis, and exosomes, DAMPs significantly influence immune regulation, thereby affecting male fertility. Understanding these roles offers new therapeutic avenues targeting DAMPs to improve male reproductive health and treat infertility.

## 1 Introduction

Male infertility is a condition characterized by the inability to achieve pregnancy after a year of unprotected intercourse (1). It is a significant cause of reproductive challenges, affecting a man’s ability to father children naturally (2). The condition can result from various factors, including genetic abnormalities (3), hormonal imbalances (4), lifestyle choices (5), and environmental exposures (6). The impact of male infertility is profound, often leading to prolonged periods of attempting conception and requiring medical interventions such as assisted reproductive technologies.

Damage-Associated Molecular Patterns (DAMPs) are endogenous molecules released by stressed or damaged cells (7). They play a crucial role in the body’s inflammatory response by activating the innate immune system (8). When released, DAMPs bind to pattern recognition receptors (PRRs) on immune cells, triggering a cascade of inflammatory processes (9). This response, while protective in the context of acute injury, can become detrimental if chronic, leading to tissue damage and impaired function (10). This review aims to explore the role of DAMPs in the male reproductive process, from spermatogenesis to fertilization, and discuss potential therapeutic strategies targeting these molecular patterns. Understanding the connection between DAMPs and the male reproductive process could provide novel insights into the mechanisms underlying spermatogenesis and fertilization, and pave the way for innovative treatments.

This narrative review employed systematic literature retrieval from PubMed and Web of Science databases using keywords: DAMPs, male infertility, spermatogenesis, sperm maturation, fertilization, and associated terms. Inclusion criteria prioritized: (1) original research elucidating DAMPs’ mechanistic roles in male reproduction; (2) clinical/animal studies linking DAMPs to sperm parameters; (3) peer-reviewed publications in English. Final analysis integrated 145 studies emphasizing the role of DAMPs in male reproduction.

## 2 Types and release mechanisms of DAMPs

### 2.1 Major types of DAMPs

The immune system’s ability to distinguish ‘self’ from ‘non-self’ is fundamental in initiating immune responses against pathogens (11). While innate immune cells use PRRs like Toll-like receptors (TLRs) to detect pathogen-associated molecular patterns (PAMPs), they also recognize DAMPs released from stressed or damaged cells (12). DAMPs activate innate immune cells, including neutrophils (13), macrophages (14), and dendritic cells (15), leading to the release of cytokines and chemokines that trigger adaptive immune responses. DAMPs also stimulate non-immune cells such as epithelial (16), endothelial (17), and fibroblast cells (18), causing them to release inflammatory mediators. Major DAMPs include but are not limited to HMGB1, HSPs, ATP, extracellular cold-inducible RNA-binding protein (eCIRP), histones, extracellular RNAs (exRNAs), cell-free DNA (cfDNA), and uric acid (19). These molecules are detected by PRRs such as TLRs, NOD-like receptors (NLRs), and RIG-I-like receptors (20). Upon DAMP recognition, TLRs activate downstream signaling pathways involving myeloid differentiation primary response 88 (MyD88) (21) and TIR-domain-containing adapter-inducing interferon-β (TRIF) (22), which in turn activate transcription factors like activator protein-1 (AP-1) (23) and nuclear factor kappa B (NF-κB) (24). Additionally, DAMPs can signal through receptors like RAGE and P2X7 (25), further propagating inflammatory responses. This complex network of signaling pathways underscores the pivotal role of DAMPs in modulating immune and inflammatory responses.

### 2.2 Release mechanisms of DAMPs

**Figure 1:**
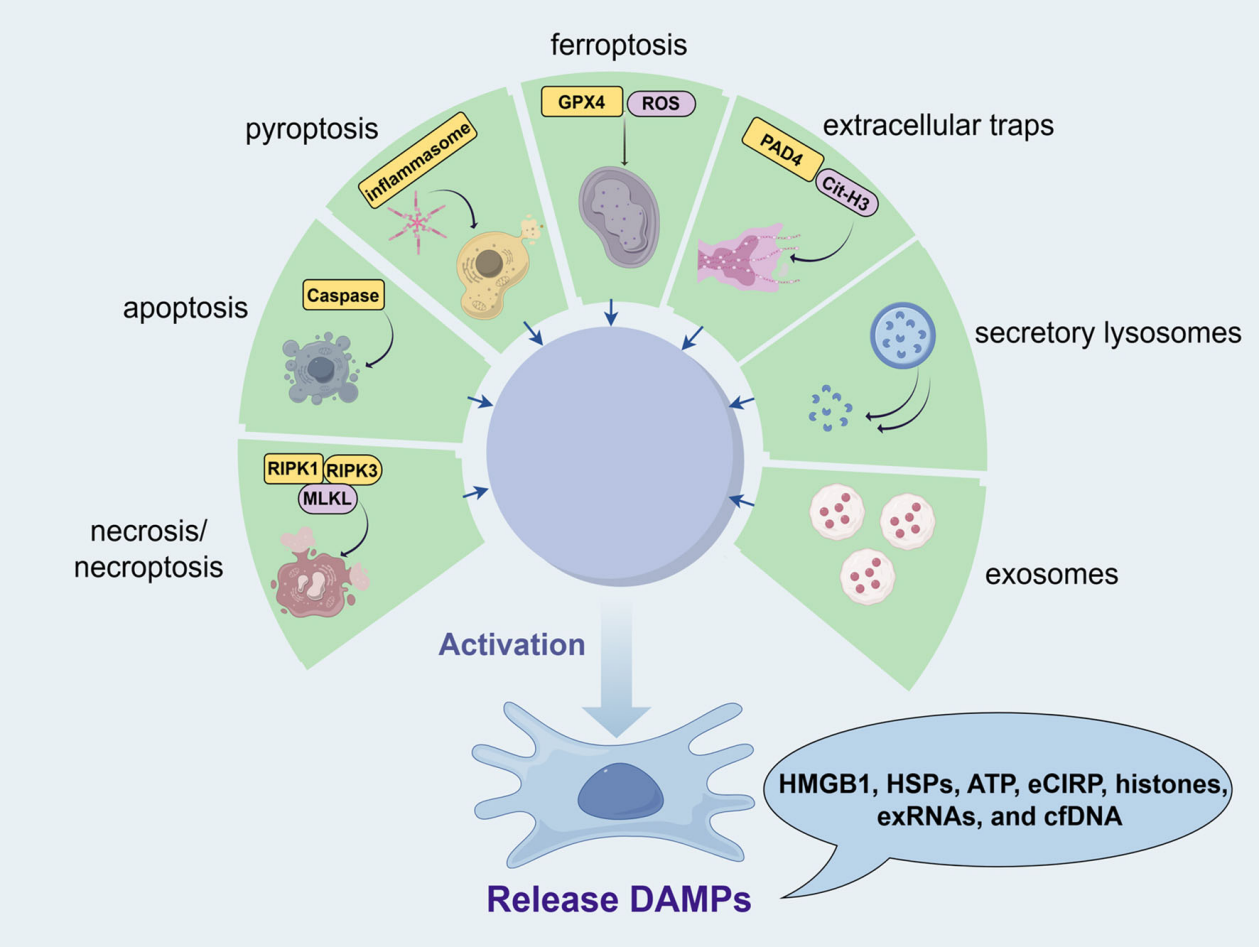
Release mechanisms of DAMPs in male reproductive health (from Figdraw, www.figdraw.com). Various forms of cellular stress, including necrosis/necroptosis, apoptosis, pyroptosis, ferroptosis, and immune responses, contribute to the release of DAMPs such as HMGB1, HSPs, ATP, eCIRP, histones, exRNAs, and cfDNA. Necrosis and necroptosis result in the uncontrolled release of intracellular contents due to cell membrane rupture, involving RIPK1, RIPK3, and MLKL. Apoptosis allows for a controlled release of DAMPs through apoptotic bodies mediated by caspase activity. Pyroptosis, driven by inflammasome activation, leads to DAMP release via gasdermin (GSDMD) pore formation. Ferroptosis, characterized by lipid peroxidation involving GPX4 and ROS, also releases DAMPs. Additionally, immune cells can release DAMPs through extracellular traps (e.g., Cit-H3) or via secretory lysosomes and exosomes, facilitating intercellular communication.

Building on the descriptions of the various types of DAMPs, their release mechanisms are diverse and complex, encompassing necrosis/necroptosis (26), apoptosis (27), pyroptosis (28), ferroptosis (29), extracellular traps (30), secretory lysosomes (7), and exosomes (31). Each of these mechanisms contributes to the release of DAMPs under different pathological and physiological conditions. For instance, necrosis and necroptosis typically result in the uncontrolled release of intracellular contents, including DAMPs, due to cell membrane rupture (32). In contrast, apoptosis generally involves a more controlled release of apoptotic bodies containing DAMPs (33). Pyroptosis and ferroptosis also contribute to DAMPs release through distinct forms of regulated cell death characterized by inflammatory responses and lipid peroxidation, respectively (34). Additionally, DAMPs can be actively secreted through extracellular traps formed by immune cells, as well as through secretory lysosomes and exosomes (35), which are specialized vesicles that facilitate intercellular communication (**Figure 1**). The specific release mechanisms and the tissue types involved in these processes are detailed in **Table 1**. These DAMP release pathways, though general in cellular biology, are also active in male reproductive tissues such as the testes and epididymis. The physiological consequences of these mechanisms—including their effects on sperm development, motility, and function—are discussed in detail in Section 4.

**Table 1:** Mechanisms and associated molecules involved in the release of DAMPs.

DAMPs	Mechanisms of release	Pathological model
HMGB1	necrosis/necroptosis (36), apoptosis (37), pyroptosis (38), ferroptosis (39), secretory lysosomes (40), exosomes (41).	Arthritis (42), Cardiovascular disease (43), Kidney injury (44), Lung (45), Cutaneous inflammation (46), Brain injury (47), Liver injury (48), Diabetes and diabetic complications (49), Parkinson’s disease (50), Macrophage polarization and inflammation (51), Ischemic stroke and hemorrhagic transformation (52), Colorectal cancer (53), Breast cancer (54), Pancreatic Cancer (55).
HSPs	necrosis/necroptosis (56), apoptosis (57), exosomes (58).	Arthritis (59), Cardiovascular disease (60), Kidney injury (61), Lung diseases (62), Cutaneous Inflammation (63), Brain injury (64), Liver injury (65), Diabetes and Diabetic Complications (66), Parkinson’s disease (67), Ischemic stroke and hemorrhagic transformation (68), Colorectal cancer (69), Breast cancer (70).
ATP	necrosis/necroptosis (71), apoptosis (72), ferroptosis (73), secretory lysosomes (74), exosomes (75).	Liver injury (76), Pre-eclampsia (77), Pancreatitis (78), Colorectum (79).
eCIRP	necrosis/necroptosis (80), secretory lysosomes (81), exosomes (81).	Sepsis (82).
Histones	necrosis/necroptosis, apoptosis (83), extracellular traps (84), exosomes (85).	Arthritis (86), Cardiovascular disease (87), Liver injury (88), Lung diseases (89), Pancreatitis (78).
exRNAs	necrosis/necroptosis (90), apoptosis (90), exosomes (91).	Cardiovascular disease (92).
cfDNA	necrosis/necroptosis (93), apoptosis (94), extracellular traps (95), exosomes (96).	Cardiovascular disease (97), Liver injury (98).
Uric acid	N/A	Liver injury (99).

N/A, The release mechanisms of Uric acid in male reproductive contexts remain uncharacterized.

## 3 Roles of DAMPs in spermatogenesis, sperm maturation and fertilization

Inflammation, along with various forms of cellular stress and damage, can significantly impact male reproductive processes, including spermatogenesis (100), sperm maturation (101), and fertilization (102). The presence of these stressors in the male reproductive tract often leads to the release of DAMPs. DAMPs play crucial roles in spermatogenesis, sperm maturation, and fertilization. Research has demonstrated the involvement of various DAMPs in different regions of the male reproductive tract, impacting these critical processes (103). Clinically, the levels of specific DAMPs have been established as valuable biomarkers, reflecting distinct infertility phenotypes (**Table 2**).

**Table 2:** Clinical evidence linking DAMPs levels in male infertility phenotypes.

DAMPs	Sample type	Clinical correlation	Key findings	Reference
HSP70 1A	Sperm	Marker of immature/abnormal sperm	Higher in abnormal sperm; may indicate failed maturation	(104)
HSP60	Seminal plasma	Subclinical genital tract infection	Significant association with leukocytospermia, elevated IL-6, IL-8, and complement C3	(105)
ATP	Seminal plasma and testicular	Impaired sperm motility	Treatment of human sperm with ATP increases fertilization rates in IVF procedures.	(106)
Histone H3	Sperm	Impaired sperm motility	Trap spermatozoa and reduce their progressive motility in a time- and dose-dependent manner.	(107)
exRNAs	Seminal Plasma	NOA Diagnosis	Biomarkers for NOA	(108)
cfDNA	Seminal Plasma​	NOA Diagnosis	Significantly elevated in NOA patients​​ vs. fertile controls	(109)
cfDNA	Seminal Plasma​	Sperm Fertility Parameters​	As biomarkers negatively correlated with sperm motility	(110)
cfDNA	​​Seminal Plasma	Non-Invasive Diagnosis of Azoospermia​	Significantly elevated in NOA patients​​ vs. fertile controls	(111)
cfDNA	Seminal Plasma	Diagnosis of abnormal spermatozoa	As biomarkers for azoospermia, teratozoospermia and sperm DNA fragmentation	(112)
Uric acid	Seminal Plasma	Diagnosis of abnormal spermatozoa	The uric acid level in the seminal fluid of infertile men is low	(113)

### 3.1 Spermatogenesis

**Figure 2:**
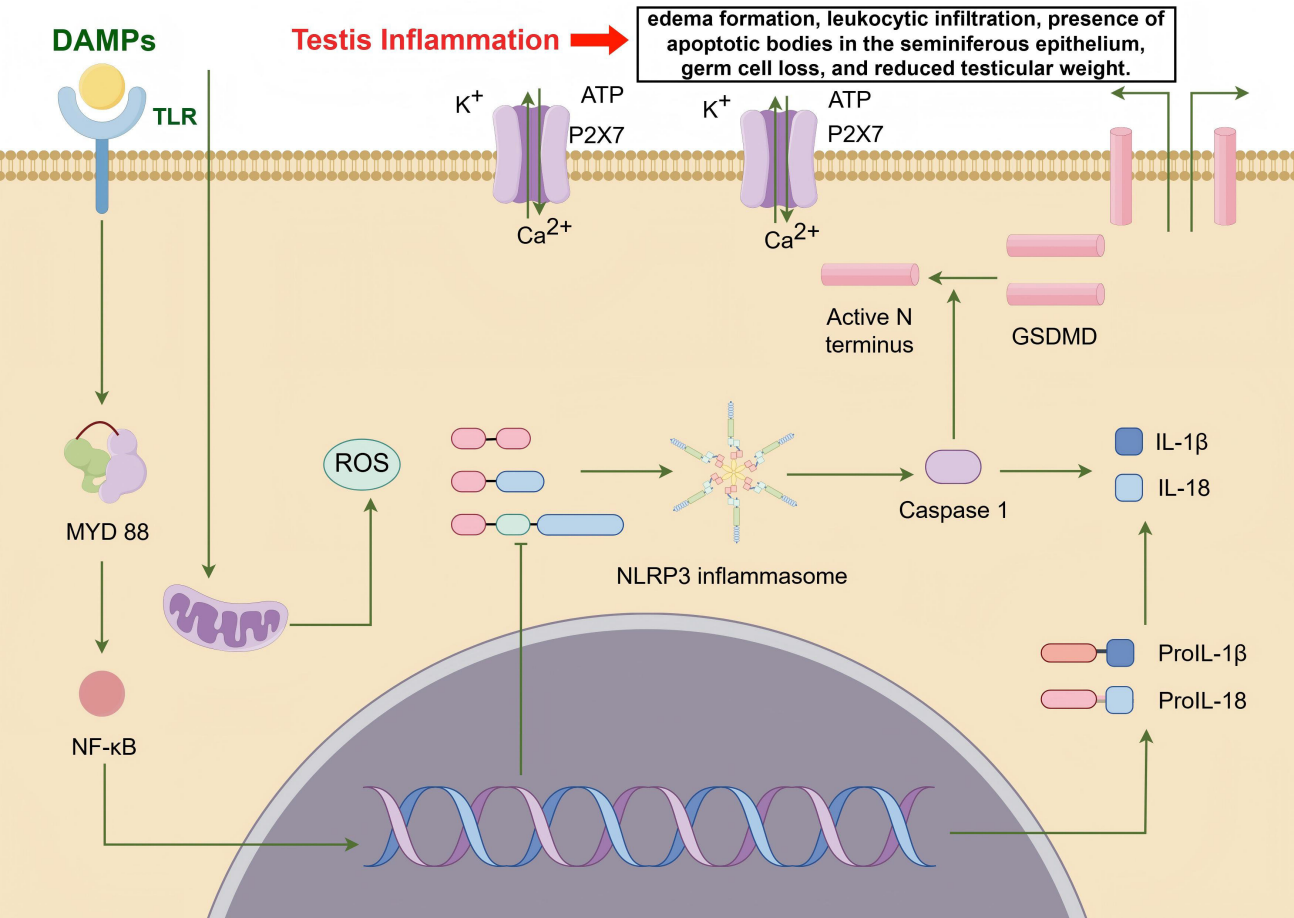
DAMPs promote testicular inflammation by regulating spermatogenesis through the activation of the inflammasome pathway (IP), particularly the NLRP3 inflammasome (from Figdraw, www.figdraw.com). DAMPs released by testicular cells activate the NLRP3 inflammasome via the TLR/MYD88/NF-κB signaling pathway, leading to the release of pro-inflammatory cytokines such as IL-1α, IL-1β, and TNF-α. Additionally, the NLRP3 inflammasome enhances the expression of Caspase-1 and IL-1β. Furthermore, DAMPs induce mitochondrial damage within cells, resulting in ROS accumulation and ATP efflux, thereby exacerbating testicular inflammation.

DAMPs play a critical role in regulating spermatogenesis and have been implicated in various mechanisms leading to male infertility. Notably, DAMPs regulate spermatogenesis by activating the inflammasome pathway (IP), particularly the NLRP3 inflammasome (114). In patients with varicocele (VCL)-associated infertility, DAMPs released by testicular cells activate the NLRP3 inflammasome, leading to the release of pro-inflammatory cytokines such as IL-1α, IL-1β, and TNF-α, which are closely associated with abnormal sperm production (115). Studies have shown that the NLRP3 inflammasome is activated in the testes of VCL patients, resulting in significantly increased levels of Caspase-1 and IL-1β, further highlighting the crucial role of the inflammasome in male infertility (116). Within the testicular microenvironment, DAMPs activate immune responses primarily through testicular macrophages, triggering robust TLR/NF-κB signaling and resulting in the secretion of pro-inflammatory cytokines and ROS, which directly contribute to spermatogenic damage (117). Moreover, HMGB1, a prototypical DAMP, also plays a significant role in spermatogenesis. In an *in vivo* rat model, linagliptin protects against cadmium-induced testicular injury by inhibiting the HMGB1/TLR4 pathway, reducing testicular inflammation, and improving sperm quantity and motility (118). Linagliptin further mitigates testicular damage by suppressing the ​​HMGB1/TLR4/NLRP3 inflammasome axis​​, leading to decreased caspase-1 activity and reduced release of pro-inflammatory cytokines ​​IL-1β and IL-18​​. This inhibition is associated with attenuated testicular cell apoptosis and enhanced autophagy flux (118). Studies have shown that mice lacking Hmgb2 (which belongs to the same HMGB family as Hmgb1) exhibit reduced fertility and impaired spermatogenesis (119), further emphasizing the importance of HMGB family proteins in spermatogenesis. Although Hmgb1 is not directly mentioned in the text, its homology suggests it may have similar effects. In experimental autoimmune orchitis (EAO), HMGB1 translocates from testicular cells, and its action can be blocked by ethyl pyruvate (EP), which reduces disease progression and spermatogenic damage (120). Excessive expression of HMGB1 in testicular cells is associated with inflammation and impaired spermatogenic function. Specifically, HMGB1 activates TLR4 on testicular macrophages, driving p38 MAPK/NF-κB-dependent production of TNF-α and IL-6, and further promoting ROS release, thereby amplifying immune-mediated spermatogenic disruption (120). In an *in vivo* rat model, high-fat diet increased testicular HMGB1 and NLRP3 levels, impairing spermatogenesis, while zinc supplementation reduced HMGB1 expression and improved sperm quantity and motility (121). Additionally, eugenol can alleviate torsion/reperfusion injury (IRI)-induced testicular damage by inhibiting the HMGB1/NF-κB axis and endoplasmic reticulum stress (122). HSPs are essential for proper spermatogenesis. For example, the conditional deletion of Hspa5 leads to spermatogenesis failure and infertility in mice (123). Specific roles of HSP isoforms in protecting sperm cells from stress and apoptosis have been detailed in various studies (124). Hyperthermia-induced stress and the expression of HSP27 are linked to disruptions in spermatogenesis and male fertility (125). Extracellular ATP plays multiple roles in sperm function, impacting both spermatogenesis and fertilization processes (126). ATP signaling in peritubular cells drives testicular sperm transport, showcasing its crucial role (127). eCIRP plays a crucial role in spermatogenesis, particularly under heat stress conditions, where its expression is downregulated, leading to impaired germ cell function (128). Studies have shown that CIRP, as a molecular chaperone, can protect germ cells from oxidative stress and apoptosis, which is especially important during testicular torsion/detorsion (129). Reduced CIRP expression is associated with varicocele and heat-induced infertility (130), suggesting that upregulating CIRP expression may be a new approach to treating male infertility. Lastly, cfDNA holds potential as a biomarker for reproductive health (131), further emphasizing the broad significance of DAMPs in spermatogenesis and male fertility (**Figure 2**). While rodent models consistently demonstrate HMGB1/NLRP3-driven spermatogenic impairment in cadmium/HFD exposure contexts, human clinical evidence remains predominantly correlative, constrained by two critical limitations: most human studies measure DAMP concentrations in semen without establishing causal mechanisms, and translational gaps persist as murine-targeted NLRP3 inhibitors lack validation in human male infertility trials.

### 3.2 Sperm maturation

Studies suggest that the role of DAMPs in sperm maturation is still relatively underexplored, but existing research has highlighted their critical functions in the epididymis. HSPs exhibit differential expression in the epididymis and play a pivotal role in sperm maturation. For instance, it has been observed that HSPs are differentially expressed in the testis, epididymis, and vas deferens of domestic cats (*Felis catus*), indicating that HSPs may have specific regulatory functions during sperm maturation (103). Furthermore, the seasonal variations in HSP concentrations in the epididymides of roe deer (*Capreolus capreolus*) further support the regulatory role of HSPs in the process of sperm maturation (132). In addition, ATP also plays a significant role in sperm maturation. Research indicates that extracellular ATP significantly affects mammalian sperm physiology (133). The purinergic signaling pathways are crucial for the recruitment of V-ATPase to the apical membrane of acidifying epididymal clear cells, which is essential for sperm maturation (134). Moreover, studies have shown that extracellular ATP and dibutyryl cAMP can significantly enhance the freezability of rat epididymal sperm (135). Overall, although research on DAMPs in sperm maturation is limited, existing evidence suggests they play crucial roles in this process. Despite the established regulatory role of DAMPs in epididymal sperm maturation, contradictory evidence persists for ATP’s function: rodent models demonstrate enhanced maturation via P2X7 receptor activation, yet human sperm exhibit reduced motility at elevated ATP concentrations (>1mM) owing to Ca²^+^-mediated toxicity. This species-specific divergence underscores an urgent need for direct human epididymal tissue investigations.

### 3.3 Fertilization

DAMPs play a crucial role in the fertilization process, influencing sperm function and the interaction between sperm and oocyte. Studies have shown that HMGB1 is a key DAMP, and its levels in follicular fluid are associated with outcomes in *in vitro* fertilization (IVF) and intracytoplasmic sperm injection (ICSI) cycles. Higher levels of HMGB1 in follicular fluid are correlated with better fertilization outcomes, indicating that HMGB1 plays an important role in the fertilization process by modulating the inflammatory response (136). HSPs, particularly HSP70 and HSP90, are also critical during fertilization. HSP70 helps maintain sperm quality and function by stabilizing the sperm plasma membrane during cryopreservation (137). Additionally, the presence of HSPs in the female reproductive tract provides a protective environment for sperm, aiding in improving fertilization rates (138). Extracellular ATP significantly affects sperm function through various mechanisms. ATP activates purinergic receptors on sperm, increasing intracellular calcium levels and thereby enhancing sperm motility, which is crucial for successful fertilization (126). Treating sperm with extracellular ATP can improve fertilization rates in IVF, especially in cases of male factor infertility (139). ATP also promotes the acrosomal reaction in bovine sperm through P2 receptors, enhancing fertilization capability (140). Moreover, extracellular ATP shows a synergistic effect on the post-thaw quality and fertilization potential of Lohi ram sperm (141). In porcine sperm, surface ATP is essential for fertilization, linked to sperm proteasomal function (142). In human sperm, ATP significantly enhances sperm motility and fertilization potential (143). Histones also have a significant impact on fertilization. Studies have found that components of neutrophil extracellular traps (NETs) adversely affect bovine sperm function, indicating the importance of histones in sperm defense mechanisms (144). Inhibiting SOCE can reduce the formation of neutrophil extracellular traps induced by human sperm, thereby improving sperm motility (145). In porcine sperm, NETs entangle sperm and embryos, hindering the fertilization process (146). Additionally, leukocytes coincubated with human sperm trigger classic neutrophil extracellular trap formation, reducing sperm motility (107). cfDNA has also gained attention for its role in fertilization. Studies indicate that cfDNA levels can serve as biomarkers for embryo quality and are associated with IVF success rates (147). High-quality embryos usually exhibit lower cfDNA levels in follicular fluid and embryo culture media (148). Furthermore, cfDNA can influence maternal immune response, potentially affecting embryo implantation and development. Elevated levels of cfDNA are associated with lower pregnancy rates (147). Uric acid’s role in fertilization has also been studied. Elevated serum uric acid levels in women with polycystic ovary syndrome (PCOS) undergoing IVF or ICSI cycles are associated with adverse reproductive outcomes, suggesting that uric acid levels may impact follicular fluid metabolic characteristics, thereby affecting fertilization and embryo development (149). A significant clinical-translational disconnect persists: while HMGB1 levels in follicular fluid correlate with improved IVF outcomes, no therapeutics currently exist to modulate oviductal DAMPs due to major barriers such as ethical constraints in manipulating human reproductive tracts and unreplicated animal findings—exemplified by porcine NETs severely impairing fertilization versus negligible effects in bovine models.

## 4 The impact of DAMPs-mediated inflammatory responses on sperm function

Studies have shown that DAMPs-mediated inflammatory responses, including necrosis, necroptosis, apoptosis, pyroptosis, and ferroptosis, have significant impacts on sperm function. During necrosis and necroptosis, the release of DAMPs such as HMGB1 triggers inflammatory responses that negatively affect sperm viability. In particular, necroptosis involves the release of DAMPs like HMGB1, which exacerbates inflammation through the TLR4 and RAGE pathways, compromising the integrity and function of sperm cells (118). On the other hand, studies have shown that reducing ATP levels can induce apoptosis or necrosis of spermatogonia (150). Apoptotic or necrotic cells can release cfDNA fragments, which negatively regulate the quality of embryos after ICSI (151). A study on normozoospermic and non-normozoospermic human samples indicated that HSP-70 expression is lower under normal conditions compared to abnormal conditions, suggesting that HSP-70 may respond to any stressor in non-normozoospermic patients. It can be inferred that HSP has anti-apoptotic effects, inhibiting the clearance of abnormal sperm cells and impairing sperm parameters (152). Additionally, in an *in vivo* rat model, Hany H. Arab et al. demonstrated that linagliptin inhibits the testicular HMGB1/TLR4/NLRP3 pro-inflammatory axis and apoptosis, thereby attenuating cadmium-induced testicular damage (118). Pyroptosis, an inflammatory form of cell death, is also a major pathway for DAMPs production. A study on semen samples from infertile patients with bilateral varicocele revealed that ROS exposure affects pathways related to pyroptosis and ferroptosis in human sperm, leading to decreased semen quality. Elevated levels of HSP90 in semen suggest a possible association with DAMPs release (153). Ferroptosis-induced ROS accumulation is related to sperm DNA damage. Increased cfDNA resulting from sperm DNA damage significantly reduces sperm fertilization ability (154). Finally, a study on a rat testicular torsion/detorsion (T/D) model found that T/D caused significant weight gain, distortion of the overall anatomical and cellular structure of the testes, poor sperm quality, redox imbalance, and inflammation in both ipsilateral and contralateral testes. This was accompanied by upregulation of xanthine oxidase/uric acid signaling and increased DNA fragmentation in the testes (155), which could be due to inflammation induced by urea and DNA release, ultimately leading to male infertility. In summary, these DAMPs-induced inflammatory responses can disrupt the environment of the testes and epididymis, negatively affecting sperm development and function. Collectively, the ‘DAMP-inflammation-sperm damage’ paradigm is predominantly established through toxin-induced rodent models. However, critical knowledge gaps persist regarding physiological DAMP functions and human disease heterogeneity. Unresolved questions include whether physiological DAMP concentrations contribute to sperm homeostasis maintenance, and why NLRP3 activation exhibits inconsistency among human varicocele patients. Resolution of these issues necessitates single-cell transcriptomic profiling of human testicular immune cells to delineate species-specific inflammatory cascades.

## 5 Conclusion

**Figure 3:**
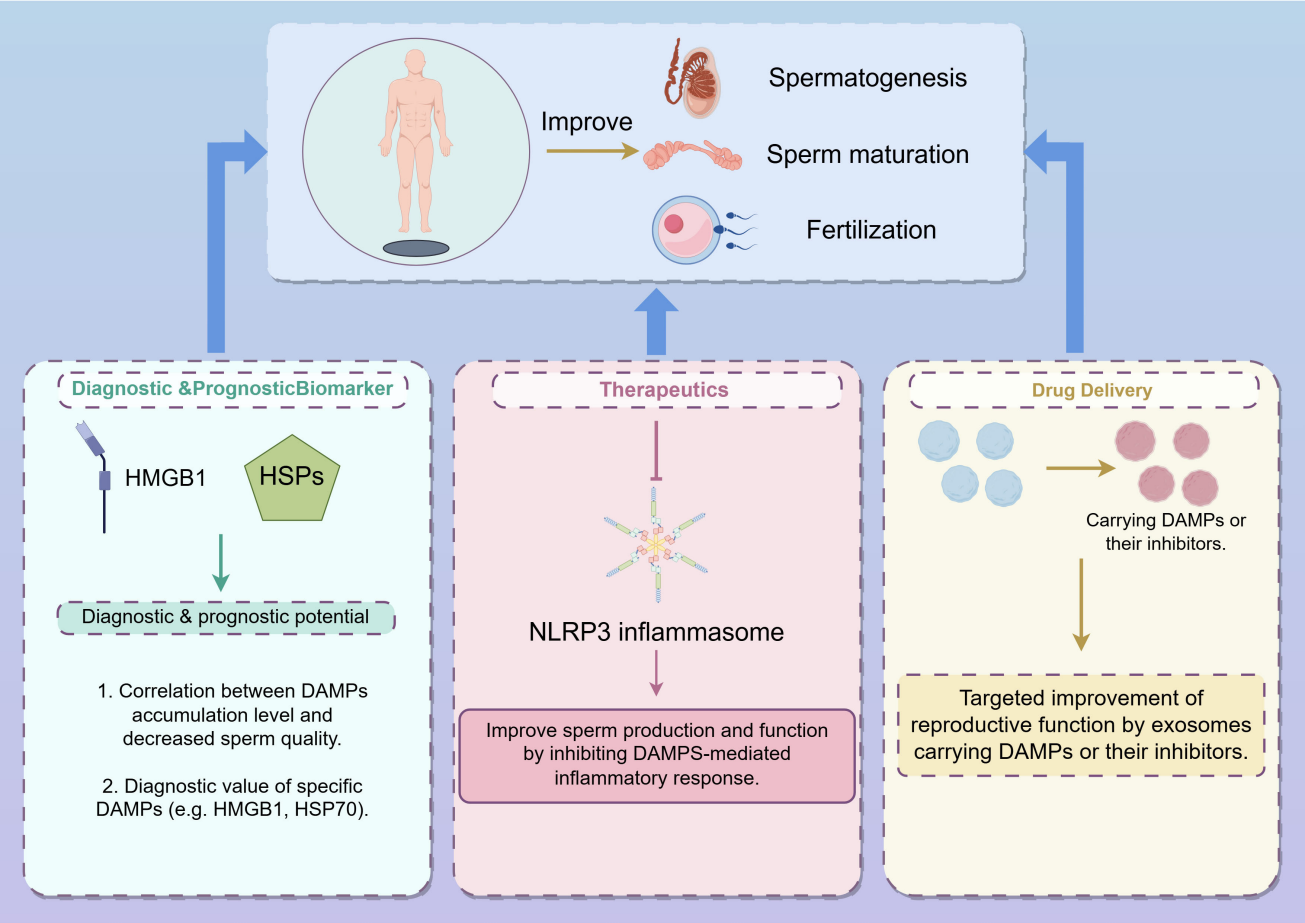
Strategies for improving spermatogenesis, sperm maturation, and fertilization through DAMPs modulation (from Figdraw, www.figdraw.com). This figure illustrates three main approaches to enhance male reproductive processes by targeting DAMPs: Diagnostic & Prognostic Biomarkers: DAMPs, such as HMGB1 and HSPs, are explored as potential diagnostic and prognostic markers for male infertility. The correlation between the accumulation of specific DAMPs and decreased sperm quality highlights their diagnostic value, particularly for DAMPs like HMGB1 and HSP70. Therapeutics: Targeting the NLRP3 inflammasome represents a therapeutic approach to improve sperm production and function. By inhibiting DAMPs-mediated inflammatory responses, it is possible to mitigate the negative effects of inflammation on male fertility. Drug Delivery: Exosomes carrying DAMPs or their inhibitors are depicted as a novel drug delivery system aimed at enhancing reproductive function. This targeted approach leverages the natural intercellular communication properties of exosomes to deliver therapeutic agents directly to the site of action, improving the efficacy of treatments for male infertility.

In this review, we have comprehensively discussed the role of damage-associated molecular patterns (DAMPs) in male infertility, highlighting their critical involvement in the pathogenesis of this condition. Our analysis reveals that DAMPs, through their diverse interactions with cellular and molecular pathways, significantly impact spermatogenesis and sperm function. Specifically, we have elucidated how DAMPs contribute to the disruption of normal sperm development and functionality, thereby exacerbating male infertility (**Figure 3**).

Our discussion illustrates the critical role of DAMPs in male infertility, emphasizing their potential as both biomarkers and therapeutic targets. The accumulation of DAMPs in the male reproductive tract and their effects on sperm quality and function present substantial implications for understanding the pathophysiology of male infertility. Targeting specific DAMPs or their signaling pathways could provide novel therapeutic avenues for managing and potentially reversing infertility conditions. Future research should focus on elucidating the precise molecular interactions between DAMPs and reproductive cells, as well as identifying specific DAMPs that could serve as reliable diagnostic markers or therapeutic targets. Investigations into how these molecules contribute to immune responses and cellular stress in the context of male infertility are crucial. Additionally, the development of targeted therapies aimed at modulating DAMPs could offer new strategies for improving treatment outcomes. In summary, the role of DAMPs in male infertility represents a promising field of research with significant clinical potential. Advancing our understanding of these mechanisms and their implications for male reproductive health will be instrumental in developing effective diagnostic and therapeutic strategies.
